# Exposure of Insects to Radio-Frequency Electromagnetic Fields from 2 to 120 GHz

**DOI:** 10.1038/s41598-018-22271-3

**Published:** 2018-03-02

**Authors:** Arno Thielens, Duncan Bell, David B. Mortimore, Mark K. Greco, Luc Martens, Wout Joseph

**Affiliations:** 1 Department of Information Technology, Ghent University - imec, Ghent, B-9052 Belgium; 2 Department of Electrical Engineering and Computer Sciences, University of California Berkeley, Berkeley Wireless Research Center, Berkeley, CA 94704 USA; 3 Department of Science and Technology, Faculty of Health and Science, University of Suffolk, Ipswitch, IP30AQ United Kingdom; 4 Newbourne Solutions Ltd, Newbourne, Woodbridge IP12 4NR United Kingdom; 5 Charles Sturt University, Medical Imaging, SDHS, Faculty of Science, Wagga Wagga, NSW 2678 Australia

## Abstract

Insects are continually exposed to Radio-Frequency (RF) electromagnetic fields at different frequencies. The range of frequencies used for wireless telecommunication systems will increase in the near future from below 6 GHz (2 G, 3 G, 4 G, and WiFi) to frequencies up to 120 GHz (5 G). This paper is the first to report the absorbed RF electromagnetic power in four different types of insects as a function of frequency from 2 GHz to 120 GHz. A set of insect models was obtained using novel Micro-CT (computer tomography) imaging. These models were used for the first time in finite-difference time-domain electromagnetic simulations. All insects showed a dependence of the absorbed power on the frequency. All insects showed a general increase in absorbed RF power at and above 6 GHz, in comparison to the absorbed RF power below 6 GHz. Our simulations showed that a shift of 10% of the incident power density to frequencies above 6 GHz would lead to an increase in absorbed power between 3–370%.

## Introduction

Radio-Frequency (RF) electromagnetic fields (EMFs) enable wireless communication between billions of users worldwide. Presently, this mainly occurs at RF frequencies located between 100 MHz and 6 GHz^1^. Wireless telecommunication base stations are the dominant sources of outdoor RF-EMFs^1^. Humans and animals alike are exposed to these fields, which are partially absorbed by their bodies, e.g. reported for insects in^2^. The absorbed dose depends on the frequency^3,4^, and can be strongly enhanced when a full-body or partial-body resonance occurs^3^. This RF absorption has already been studied for particular insects at different individual frequencies: 27 MHz^5,6^, 900–915 MHz^6–8^, and 2450 MHz^9^.

This absorption may cause dielectric heating^10^. Heating affects insect behavior, physiology, and morphology^11^. Reviews of studies that investigate RF heating of insects are presented in^12–14^. Other authors focus on environmental RF exposure of insects^15,16^ or expose insects to RF radiation in order to investigate potential biological effects^17,18^. Studies on non-thermal effects of exposure to RF-EMF exist:^19^ presents a review of potential mechanisms for non-thermal effects and a review of non-thermal effects of EMF exposure wildlife is presented in^20^. Most existing studies focus on RF frequencies below 6 GHz. The same frequencies at which the current generations of telecommunication operate^1^. However, due to an increased demand in bandwidth, the general expectation is that the next generation of telecommunication frequencies will operate at so-called millimeter-wavelengths: 30–300 GHz^21,22^. Therefore, future wavelengths of the electromagnetic fields used for the wireless telecommunication systems will decrease and become comparable to the body size of insects and therefore, the absorption of RF-EMFs in insects is expected to increase. Absorption of RF energy was demonstrated in insects between 10–50 GHz^23^, but no comparison was demonstrated with the RF absorption at frequencies below 10 GHz. The radar cross section of insects has been determined above 10 GHz, but this quantity includes both scattering and absorption^24^. It is currently unknown how the total absorbed RF power in insects depends on the frequency to which they are exposed.

Most of the previously cited studies depend on measurements using RF equipment such as antennas, waveguides, and dielectric probes to determine the absorption of RF-EMFs in insects. An alternative approach would be to use numerical simulations. This approach was previously used to determine the absorption of RF-EMFs in humans and requires numerical models or phantoms^25–28^.

Techniques for creating heterogeneous, three-dimensional insect models with micrometer resolution have previously been demonstrated in^29^.

However, up to now, insect phantoms have not been used in electromagnetic simulations.

The aims of this study were to, for the first time, numerically evaluate RF-EMF absorption in real models of insects and to determine a potential difference in RF absorption in insects due to current and future telecommunication networks. To this aim, we studied the absorbed RF power in four different insect models obtained using micro-CT imaging as a function of frequency in a broad band, 2 GHz up to 120 GHz, that covers both the existing and the foreseen future wireless telecommunication bands. Voxelling precision in the order of 5–20 *μ*m is obtained, required for accurate electromagnetic simulations.

## Methods

### The Insects

#### Australian Stingless Bee (Tetragonula carbonaria)

This bee *(Tetragonula carbonaria)* is native to Australia. The scanned insect was approximately 4.5 mm long, 3.0 mm wide, and has a mass of 2.5 mg.

#### Western Honeybee (Apis mellifera)

This bee *(Apis mellifera)* originated in Europe. It is the most common honeybee. The studied specimen was approximately 11.0 mm long, 5.0 mm wide, and has a mass of 900 mg.

#### Desert Locust (Schistocerca gregaria)

The studied locust *(Schistocerca gregaria)* was approximately 55.0 mm long, 18.0 mm wide, and has an approximate mass of 3.5 g.

#### Beetle (Geotrupes stercorarius)

The studied beetle is a dor beetle *(Geotrupes stercorarius)*. The beetle was found and scanned (see below) at Aberdeen University in Scotland. The beetle’s length was 8.01 mm and its width is 4.5 mm. The insect’s mass was not measured at the time of scanning. The average mass of a dor beetle is 220 mg^30^.

### Scanning Methods

#### Australian Stingless Bee

MicroCT scans were performed with a Skyscan 1172 high-resolution MicroCT system (Bruker MicroCT, Kontich, Belgium). This system has a sealed, microfocus x-ray tube with a 5 *μ*m focal spot size. The x-rays were produced by exposing the anode to 40 kV at 100 *μ*A. Prior to scanning, the sample containing the insect was placed on the pedestal between the x-ray source and the CCD detector. After positioning the sample, 600 2D x-ray images over 180° were captured by exposing the sample and then rotating it to the next exposure position with a slice-to-slice rotation distance of 2 *μ*m, and a total acquisition time of approximately 60 min: each 2D image represents one slice. The scanner software then converted each slice to axial orientation and created 998 bitmap images (16 bit grey scale) which were stored for 2D viewing and 3D reconstruction as a 983 Mb dataset. The resulting isotropic voxel size was 5 *μ*m.

#### Western Honeybee

A bench-top MicroCT scanner (Quantum GX MicroCT Imaging System, PerkinElmer, Hopkinton, MA, USA) at the Western Sydney University National Imaging Facility (Sydney, Australia) was used to scan the bee. The following parameters were used: 50 kVp, 80 *μ*A, high resolution 2048 × 2048 pixels image matrix, with 20 *μ*m isotropic voxel size. Scanning time was 3.0 s for each of the 180 projections with 3.0 s rotation in between each projection. The total scan time was approximately 18 min per whole bee. The Quantum GX, bench-top MicroCT scanner’s software was used to reconstruct the 180 projection images and then to convert them into a 2D rendered image stack of 512, 16 bit bitmap images. Bee volume data were then acquired by loading the image stack into BeeView volume rendering software (DISECT Systems Ltd, Suffolk, UK).

#### Desert Locust

The locust was suspended vertically in a 30-mm acrylic tube that was mounted tightly on the micro-CT’s inclination stage. This stage was used to ensure that the rotation axis was at 90° to the x-ray source. Exposure factors were: 50 kVp and 198 *μ*A. The data were isotropic 16 bit 2000 × 2000 pixels with 1048 rows. Pixel size was 10.469 *μ*m. Skyscan NRecon software version 1.5.1.4 (Bruker, Kontich, Belgium) was used to reconstruct the projection data^31^. Having obtained the projection data in the form of an image stack of 2-D TIFF files the data was viewed as a 3-D model using Disect software, DISECT Systems^29^.

#### Beetle

The beetle was scanned at Aberdeen University on a Skyscan 1072 Micro-CT scanner (Bruker, Kontich, Belgium) using 50 kV and 197 *μ*A, at 10.46 *μ*m pixels isotropically. The images were then converted to axial slices using Skyscan’s NRECON software (version 1.4). The produced axial image stack was further processed and analyzed using the Tomomask software (www.tomomask.com) before viewing in disect.

### Development of 3D models

3D models of the insects were created using the software TomoMask (www.tomomask.com). The image stack for each insect was firstly imported into the software together with details of the pixel and slice spacing. Regions to be converted into a 3D model are defined in TomoMask by drawing a mask of the wanted regions on each slice. This can be done automatically using the Luminance mask function which creates a mask based on the grey level of the pixels. The threshold values for the mask are set to include all of the insect tissue but will exclude air cavities and very fine structures, such as wings. The 3D model (generated by a marching cubes algorithm^32^) is exported as an STL (STereo Lithography)^33^ format file. STL files describe only the surface geometry of a three-dimensional object without any representation of colour or texture. Typically some smoothing of the models is required and this is realized using the Taubin *λ*/*μ* smoothing scheme^34^ implemented in MeshLab^35^. The Taubin method is good at removing noise whilst preserving shapes and features. Dimensions of the models and mesh integrity are finally checked (and corrected if necessary) using Netfabb (Autodesk, San Rafael, CA, USA).

### Dielectric Properties

The propagation of EMFs inside and around the obtained 3D insect phantoms will depend on their dielectric properties: the relative permittivity (*ε*_*r*_) and conductivity (*σ*). In this study, we have executed and relied on a literature review of previous measurements of dielectric properties of insects, predominantly using the coaxial-line probe method^36^. There exist alternative methods. A toroidal resonator was used to determine the dielectric properties of two insects at 2370 MHz^37^. Dielectric properties of Rice Weevils (Sitophilus oryzae) are obtained using the coaxial probe method for frequencies from 5 × 10^4^–2 × 10^10^ Hz^2^. The same technique was used on three other insects: the Red Flour Beetle *(Tribolium castaneum)*, the Sawtooth Grain Beetle *(Oryzaephilus surinamensis)*, and the Lesser Grain Borer *(Rhyzopertha dominica)*, from 0.2–20 GHz^36^. The same method was also used to measure dielectric properties of four insects: the Codling Moth *(Cydia pomonella)*, the Indian Mealmoth *(Plodia interpunctella)*, the Mexican fruit fly *(Anastrepha ludens)*, and the Navel Orange Worm *(Amyelois transitella)* from 27–1800 MHz^6^. Coaxial measurements on a Colorado Beetle *(Leptinotarsa decemlineata)* were performed from 0.1–26.5 GHz and used to derive a fit to the measurement data^38^.

We have pooled the data series, real and imaginary part of *ε*_*r*_ as a function of frequency, obtained by^6,36,38^ and interpolated them from 2–120 GHz in steps of 0.1 GHz. We have then averaged over all available data at every frequency steps considered in the simulations.

### Numerical Simulations

**Figure 1:**
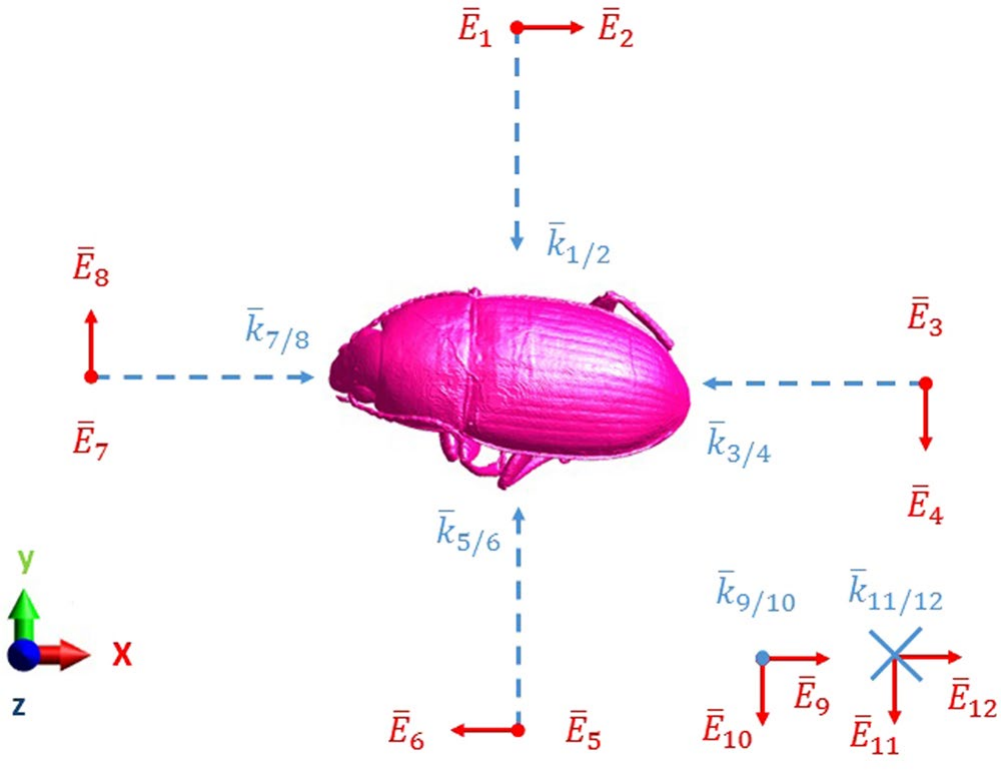
Illustration of the RF-EMF exposure set up. The insect (Beetle shown here in pink) is exposed to twelve RF plane waves incident from six directions along the positive and negative directions of the Cartesian axes shown on the bottom left with two orthogonal polarizations for each direction. The twelve wave vectors ${\overline{k}}_{i/j}$ are indicated in blue (dashed arrows), while the polarization of the incident electric fields ${\overline{E}}_{i}$ are indicated in red. *i* and *j* indicate the configuration number, from 1 to 12.

The Finite-Difference Time-Domain (FDTD) technique implemented in the commercial simulation software Sim4life (ZMT, Zurich, Switzerland) is used to evaluate absorption of RF-EMFs inside the insects as a function of frequency. This technique is commonly used to determine absorption of RF-EMF in heterogeneous human body models^3^. The FDTD method requires one to discretize the simulation domain using a three-dimensional grid. The simulation domain is divided in a number of cubes (discretized) with spatial extends that are defined by the spatial grid steps in the simulation domain. RF-EMFs can be incident from any direction. Therefore, we have chosen to work with 12 incident plane waves with a root-mean-squared electric field strength of 1 V/m, illustrated in Fig. 1, along 6 directions defined by Cartesian axes, with two orthogonal polarizations of the incident RF-EMFs along each axis.

The exposure was modeled using single frequency sinusoidal (harmonic) continuous plane waves. We did not take into account a potential modulation of the waves, which might be present in real telecommunication signals. This same technique has previously been used to evaluate the frequency dependence of RF absorption in the human body^3^. Simulations were executed for sinusoidal plane waves at 7 harmonic (single) frequencies: 2, 3, 6, 12, 24, 60, and 120 GHz. This resulted in a dataset of 4 (insects) ×7 (frequencies) ×12 (plane waves: 6 angles of incidence ×2 polarizations) = 336 simulations.

The Australian Stingless Bee, the Western Honey Bee, and the Beetle were discretized with steps of 0.05 mm in each direction, while the larger Locust was discretized with steps of 0.2 mm in each direction at frequencies below 60 GHz and a step of 0.1 mm at 60 GHz and 120 GHz. These spatial steps provided a balance between simulation time (which depends on the number of grid steps and the relative grid step size in comparison to the wavelength) and spatial resolution of the insects’ features. A stable FDTD simulation yields reproducible results that converge over time. The quantities determined using the FDTD algorithm should converge to a constant value as the simulation progresses in time. After a certain simulation time, these values will remain constant, this is referred to as a steady state. A grid step smaller than one tenth of the smallest wavelength in the simulation domain is necessary for a stable FDTD simulation^39^. This is a requirement of the FDTD algorithm^39^ and remains valid in all our simulations. The smallest wavelength in tissue $(\lambda /\sqrt{{\varepsilon }_{r}})$ is 1.1 mm at 120 GHz. At this frequency we used grid steps of 0.05 mm $(\le 0.045\times \lambda /\sqrt{{\varepsilon }_{r}})$ for all insects, except for the locust where we used 0.1 mm $(\le 0.09\times \lambda /\sqrt{{\varepsilon }_{r}})$.

We ensured that the grid steps were small enough to prevent disconnections in the models. All insects were considered as consisting of homogeneous tissue with frequency-dependent dielectric parameters obtained as an average of the values we found in literature (previous section). This is an approximation, since real insects have heterogeneous tissue properties. Each simulation was executed until a steady state was reached. The number of periods necessary to reach a steady state solution depended on the studied insect and frequency and was between 20–80. This was controlled by temporal monitoring of the electric field strength along a line in the simulation domain until it reached a steady state. Additionally, the chosen number of simulation periods allowed for propagation of at least 3 times the length of the insects’ diagonal (see Table 1).

**Table 1:** Dimensions of the studied insect models along the different axes shown in Fig. 1.

Insect	L (mm)	W (mm)	H (mm)	D (mm)	Range *λ*_*max*_ (mm)
Beetle	8.01	4.5	4.29	10.14	5–25
Australian Stingless Bee	4.89	3.39	3.99	7.16	2.5–12.5
Western Honey Bee	11	4.154	4.044	12.43	12.5–50
Locust	54.99	18.49	17.55	60.61	25–100

L, W, and H, are the dimensions in the X, Y, and Z, directions, respectively. D is the size of the diagonal of the brick with dimensions L × W × H. The final column lists the range in wavelengths where the maximal *P*_*abs*_(*λ*_*max*_) will be located.

After every simulation, the absorbed RF-EMF power (*P*_*abs*_) in the insect was extracted. The *P*_*abs*_ is calculated as the product of the conductivity and the squared electric field strength integrated over the volume of the insect. The whole-body averaged specific absorption rate can be obtained by dividing *P*_*abs*_ by the insects’ mass (assuming a homogeneous mass density). Absorbed RF-EMF power is generally used as a proxy for dielectric tissue heating^10^. We have not executed full thermal simulations due to uncertainties on the specific heat capacities of the insects and heat dissipation mechanisms.

## Results

### 3D Models

**Figure 2:**
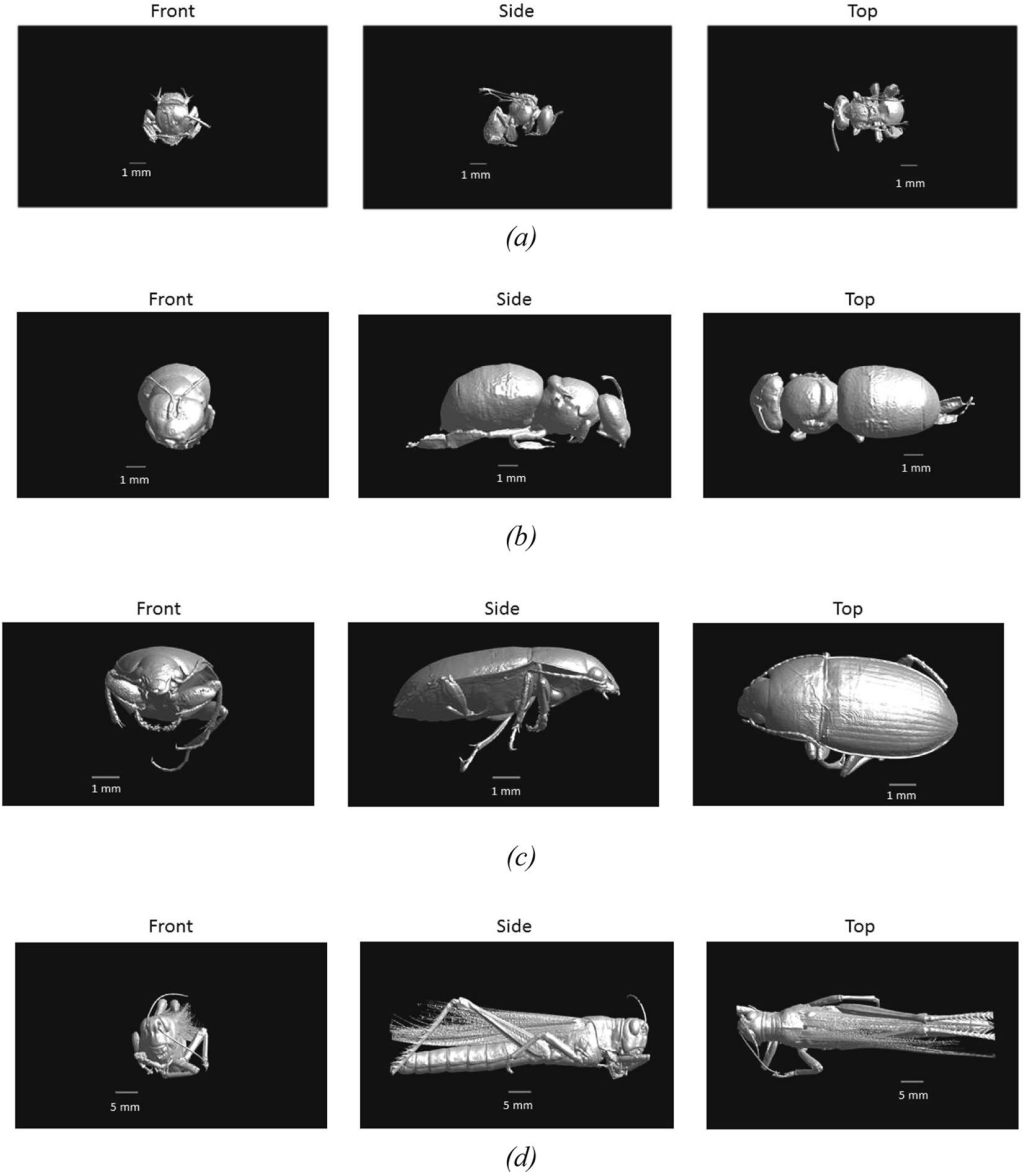
Frontal, side, and Top view of the four studied insects. (**a**) Australian Stingless Bee, (**b**) Western Honeybee, (**c**) Beetle, and (**d**) Locust.

Figure 2 shows the used 3D models obtained using micro-CT scanning of four insects.

### Dielectric Properties

**Figure 3:**
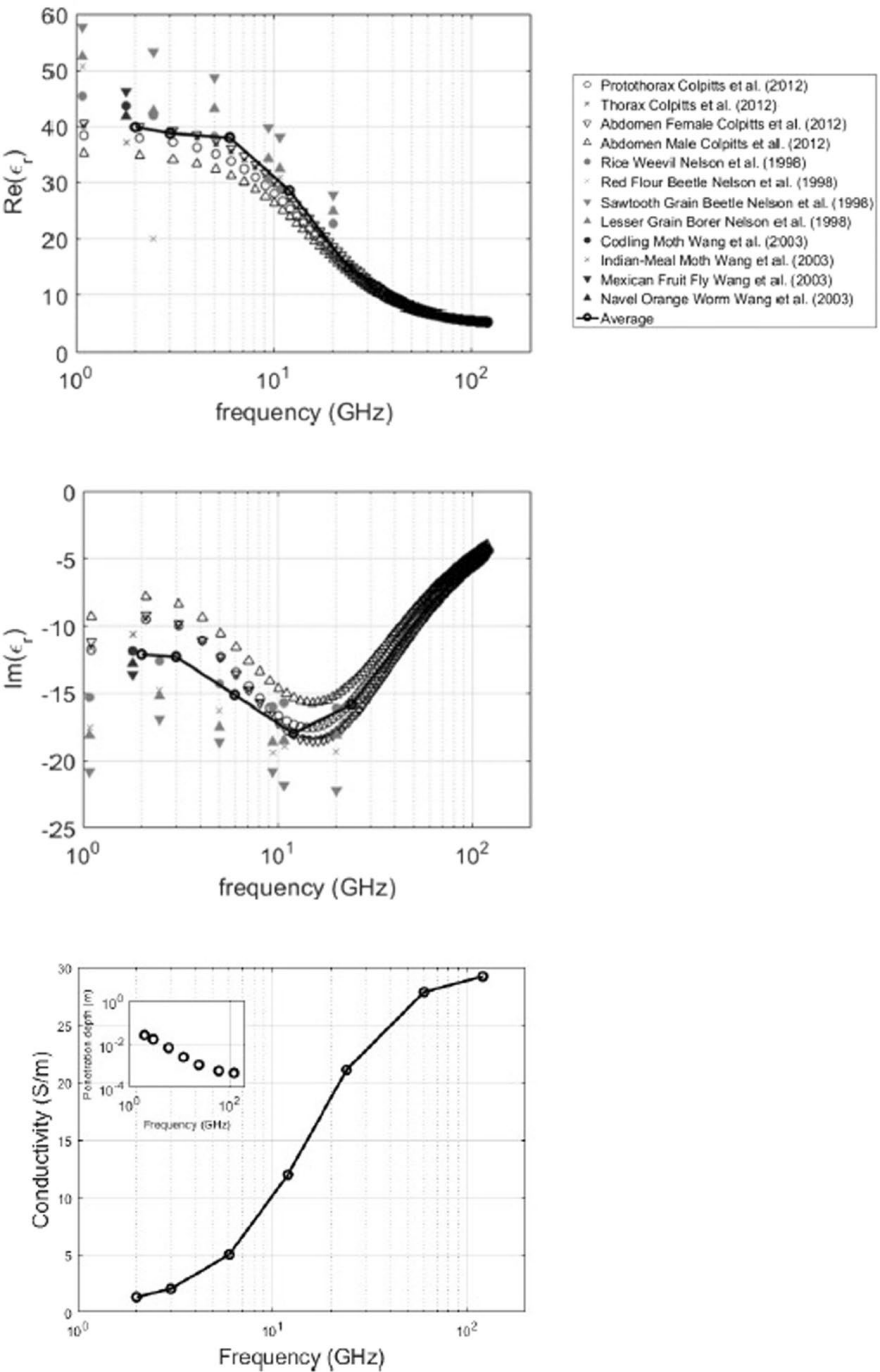
From top to bottom: Real part of the used dielectric permittivity, Imaginary part of the used dielectric permittivity, and conductivity with RF-EMF penetration depth as an inset. Markers show measurements obtained from literature. The black line with circular markers shows the average over the available data series at those frequencies.

Figure 3 shows the imaginary and real parts of *ε*_*r*_ obtained by averaging those values that were available in^6,36,38^. The real part is positive and decreases with frequency, while the imaginary part is negative (lossy media) and shows a minimum between 10–20 GHz. These are in line with the Debye dielectric curves proposed in^38^. Figure 3 adds further perspective by showing the corresponding conductivity in (S/m) and the RF penetration depth.

### Numerical Simulations

**Figure 4:**
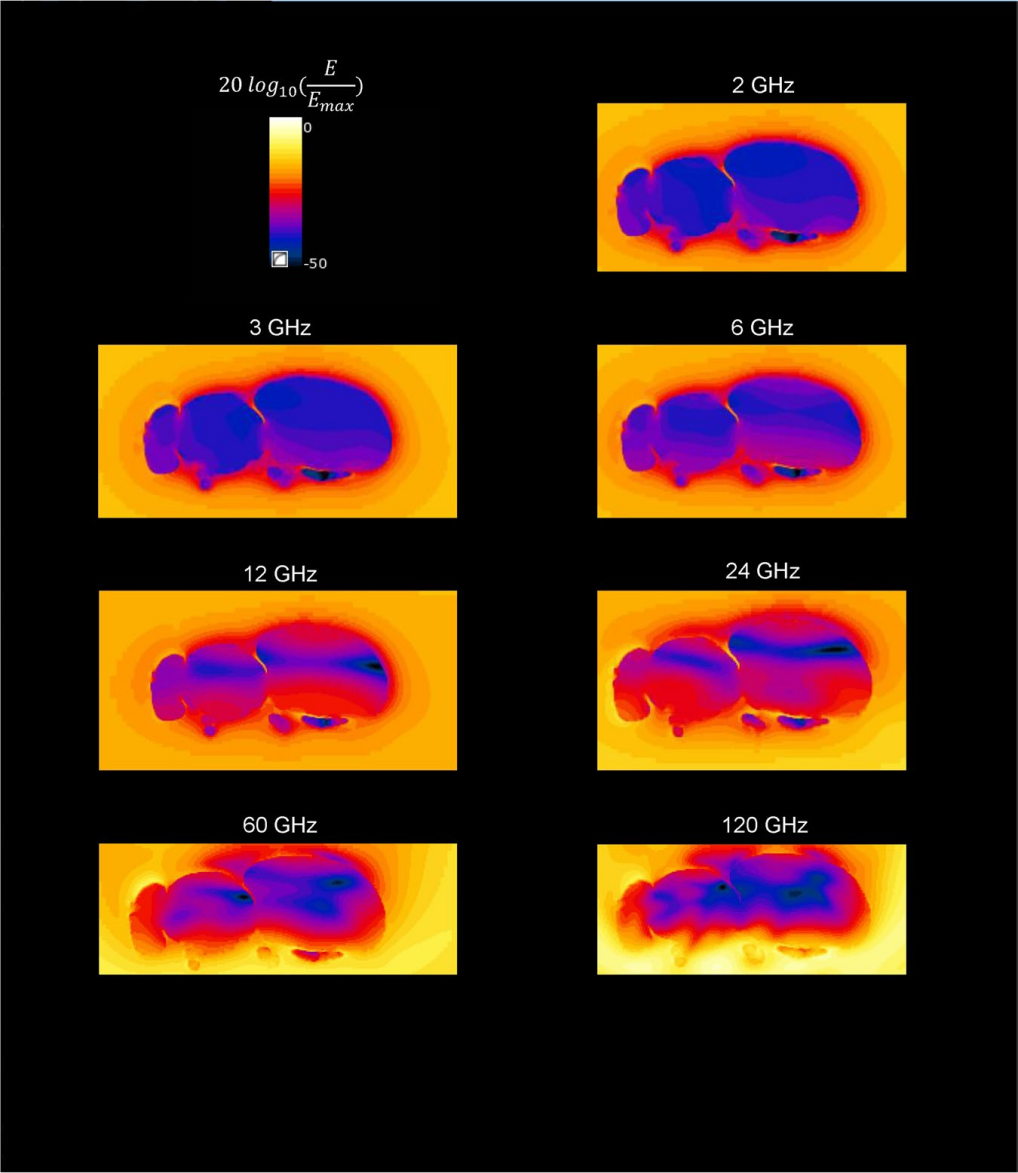
Normalized Electric field strength (dB) in a mid-transverse cross section of the Western Honey Bee as a function of frequency for a single plane wave incident from below with polarization orthogonal to the shown plane (No. 5 in Fig. 1). Normalization was executed for each simulation separately, i.e. *E*_*max*_ can be different in each subfigure.

Figure 4 illustrates the frequency dependence of the absorption of RF-EMFs in the Western Honeybee in terms of the ratio of the electric field strength inside the insect to the maximum electric field in the simulation domain. At the currently used frequencies for telecommunication (<6 GHz), the wavelength is relatively large compared to the insects and the waves do not penetrate into the insects, which results in lower Pabs values. At 12–24 GHz, the fields penetrate more and more into the insect as the wavelength becomes comparable to the insects’ size and the conductivity increases as well. At the highest studied frequencies, the fields penetrate less deep into the insect, but their amplitude is higher, resulting in a similar or slightly lower *P*_*abs*_.

**Figure 5:**
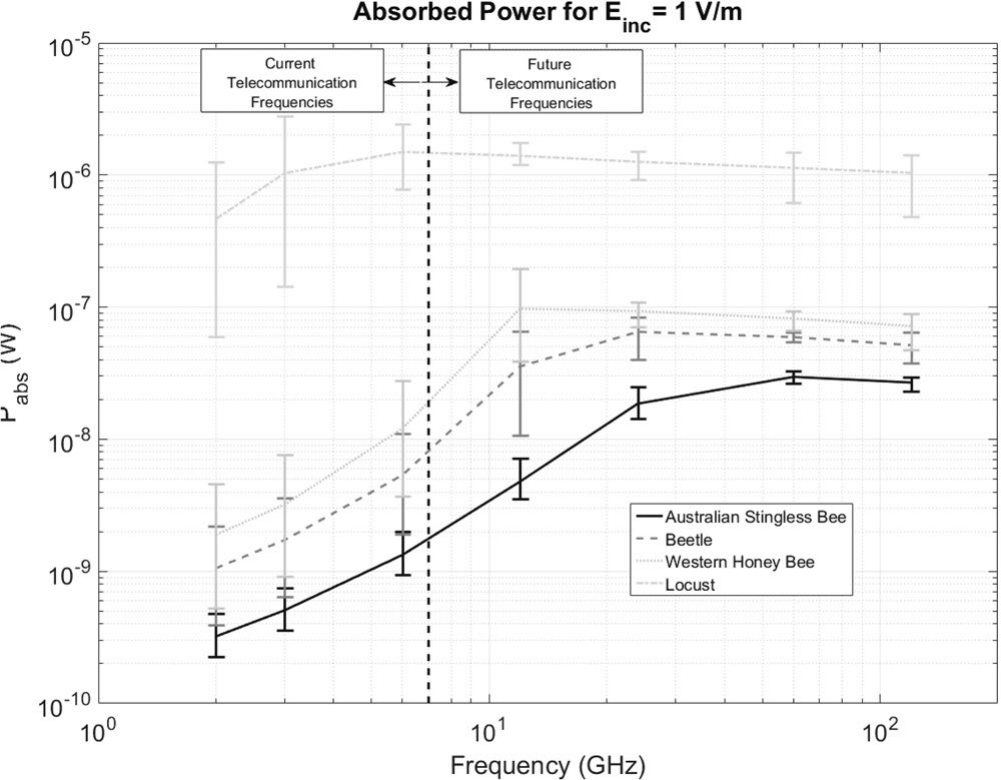
*P*_*abs*_ for an incident field strength of 1 V/m as a function of frequency for all studied insects. The markers indicate the average over all twelve plane waves at each of the simulated frequencies, while the whiskers indicate the minimal and maximal Pabs values obtained during the simulations.

Figure 5 shows the *P*_*abs*_ linearly averaged over all twelve plane waves as a function of frequency for all studied insects. The absorbed power increases with increasing frequency from 2–6 GHz for all insects under exposure at a constant incident power density or incident electric field strength of 1 V/m. The absorbed power in the Locust, the largest studied insect, decreases slightly at the studied frequencies >6 GHz, but remains higher than at 2 and 3 GHz. The Western Honeybee shows an increase up to 12 GHz, followed by a slight decrease up to 120 GHz (Pabs remains more than 10× higher than <6 GHz). The smaller Australian Stingless Bee shows an increase of *P*_*abs*_ with frequency up to 60 GHz and a slight decrease at 120 GHz. The Pabs in the Beetle increases until 24 GHz and slightly decreases at higher frequencies.

Table 1 lists the dimensions of the different studied insects, compared to the wavelength *λ*-range in which the maximal Pabs will be located. The Pabs is simulated for discrete frequency steps. Therefore, the *λ*_*max*_ that corresponds to the maximum *P*_*abs*_ is located in between the wavelength steps right below and above the wavelength step that corresponds to the maximum simulated *P*_*abs*_, see Fig. 4. The main diagonal of the insects’ bounding box is within the range in which the wavelength of maximal absorption *λ*_*max*_ is located for three out of the four studied insects. This indicates that the absorption is (partly) determined by the size of the insects.

Numerical simulations are never the same as reality and there are always uncertainties associated with any EM simulation technique. We report the following sources of uncertainty: model variations and variation on dielectric properties.

The insect models are scanned with a resolution of 20 *μ*m, 10.5 *μ*m, 10.5 *μ*m, and 5 *μ*m, for the Honey Bee, the Locust, the Beetle, and the Australian Stingless Bee, respectively. These are 40%, 5–10%, 21%, and 10% of the spatial grid step used in the simulations of the Honey Bee (0.05 mm), the Locust (0.1–0.2 mm), the Beetle (0.05 mm), and the Australian Stingless Bee (0.05 mm), respectively. This indicates that the grid step is dominant in determining the spatial extends of the used models and not the resolution of the scanning method. In order to investigate the effect of the chosen grid step on the obtained *P*_*abs*_ values, we have executed the simulation with configuration 9 (Fig. 1) at 120 GHz with a maximal grid step that is half of the grid step used in our simulations using all four studied insects. We assume the largest effect of grid step size at the highest frequency. A 50% reduction in grid step (more accurate modelling) resulted in deviations of 1.1%, 2.5%, 0.32%, and 0.24%, for the Honey Bee, the Locust, the Beetle, and the Australian Stingless Bee, respectively. These deviations are small in comparison to the variations as a function of frequency, see Fig. 5, and the uncertainty caused by the dielectric parameters, see the next paragraph.

Deviations on *ε*_*r*_ will influence *P*_*abs*_: the real part of *ε*_*r*_ will (partly) determine the magnitude of the internal electric fields, while *P*_*abs*_ scales linearly with conductivity. The maximal relative deviations on the real and imaginary part of *ε*_*r*_ are (−13, +36)% and (−40, +36)%, respectively, which occur between 2–3 GHz. We have executed a simulation using configuration 1 at 2 GHz for the Beetle phantom, shown in Fig. 1, using five different sets of dielectric properties accounting for the deviations mentioned above: [*Re*(*ε*_*r*_), *Im*(*ε*_*r*_)], [1.36 × *Re*(*ε*_*r*_), 1.36 × *Im*(*ε*_*r*_)], [1.36 × *Re*(*ε*_*r*_), 0.6 × *Im*(*ε*_*r*_)], [0.87 × *Re*(*ε*_*r*_), 1.36 × *Im*(*ε*_*r*_)], and [0.87 × *Re*(*ε*_*r*_), 0.6 × *Im*(*ε*_*r*_)], in order to determine the effect of the uncertainty of dielectric properties on *P*_*abs*_. We found maximal relative deviations of [−57, +59]% relative to the value obtained using [*Re*(*ε*_*r*_), *Im*(*ε*_*r*_)]. These deviations are small in comparison to the variations as a function of frequency, see Fig. 5.

Previous studies have indicated that large differences in dielectric properties might exist between adult insects and larvae^40^. Worst-case deviations of [*Re*(*ε*_*r*_)/7, *Im*(*ε*_*r*_)/5] at 5 *GHz* and [*Re*(*ε*_*r*_)/6, *Im*(*ε*_*r*_)/8] at 15 *GHz* were observed in^40^. We have executed simulations of configuration 1 using the beetle (shown in Fig. 1) at 6 *GHz* and 12 *GHz* where we have applied these reduced dielectric parameters. We found an increase in *P*_*abs*_ of 4% at 6 *GHz* and a decrease of 66% in *P*_*abs*_ at 12 *GHz*. Figure 5 shows that these variations are smaller than the variations we observed for varying angles of incidence at a fixed frequency.

## Discussion

In this study, we have evaluated the absorption of RF-EMFs in insects as a function of frequency. To this aim, we obtained novel insect models using micro-CT imaging, which were used in FDTD simulations. In these simulations they were exposed to plane waves incident from six directions and two polarizations.

The frequency of the incident harmonic plane waves was varied from 2–120 GHz and resulted in *P*_*abs*_ as a function of frequency.

Previous studies have shown that Micro-CT imaging can be successfully used as a non-invasive technique to accurately scan insects and develop 3D models with micrometer resolution^29,41^. Models with micrometer resolution are necessary to obtain accurate results in FDTD simulations at 120 GHz (*λ* = 2.5 mm), since a discretization of *λ*/10 in the simulation domain is recommended to obtain stable results^39^. It has been demonstrated for human body models that real anatomical models generally result in more accurate and realistic results than approximate models^3,25,28^. Therefore, we also expect our real insect models to lead to more accurate results regarding absorbed RF power than, for example, cylindrical phantoms with different diameters and heights, which were used in previous studies of RF exposure of insects^42^.

The dielectric properties that were assigned to the studied insects were obtained from an interpolation of data found in literature. Ideally, the simulations should be executed with dielectric properties measured in the actual insects that were used to create the models. Figure 3 does show that most insects show a similar frequency behavior, which we have averaged by using an interpolation over values listed in literature.

Our numerical simulations show that the absorption of RF-EMFs in the insect models is frequency dependent. The *P*_*abs*_ is smallest at the lowest studied frequencies 2 GHz and 3 GHz, for all insects. *P*_*abs*_ shows a peak, which depends on the size and/or mass of the insects. The three smaller insects show their maximum at a frequency higher than 6 GHz: 60 GHz, 24 GHz, and 12 GHz for the Australian Stingless Bee, the Beetle, and the Honey Bee, respectively. The Locust shows a maximum at 6 GHz. We attribute this frequency behavior to two effects: first, the efficiency of RF-EMFs coupling into the models is maximal at frequencies comparable to the length of the insects, as Table 1 illustrates. Second, the interpolation of the imaginary part of the dielectric constant shows a minimum at 12 GHz, which means that RF-EMFs can cause the highest local SAR at these frequencies, see Fig. 3.

The difference between the maximal and minimal P_abs_ found at one frequency for different angles of incidence is smaller at the frequencies >6 GHz, than at the frequencies <6 GHz, in particular for the three smaller insects. This indicates that the angle of incidence is less important at these frequencies. This suggests that there is little difference in efficiency when depositing RF power in the studied insects with a single plane wave compared to depositing the same power using uncorrelated sources or reflections coming from all directions. In this study, we have only used single plane-wave simulations to determine *P*_*abs*_. The averaging over *P*_*abs*_ does not include interference effects, which might result in lower (destructive interference) or higher (constructive interference) bounds on the *P*_*abs*_ values shown in Fig. 5.

A similar frequency behavior (increase, peak, decrease, and dependency on body size) is observed in human body models^3,4^. However, at frequencies which are roughly a factor 100–1000 times lower, because the human body is approximately the same order of magnitude larger than that of the studied insects. For example, the heterogeneous adult human body model Duke shows an increase in Pabs from 10 MHz–80 MHz, a peak between 80 MHz–90 MHz, followed by a decrease in P_abs_ (and a second peak at higher frequencies)^3^. The smaller child phantom Thelonius shows an increase in Pabs from 10 MHz–100 MHz, a peak between 100 MHz–200 MHz, followed by a decrease in *P*_*abs*_^3^.

In order to quantify the effect of a shift to higher telecommunication frequencies on *P*_*abs*_, one can use the data presented in Fig. 5. If we assume an incident *E*_*rms*_ = 1 V/m which is uniformly distributed over 2, 3, and 6 GHz, we find average *P*_*abs*_ values of 0.71 nW, 2.6 nW, 5.7 nW, and 990 nW, for the Australian Stingless Bee, the Beetle, the Honey Bee, and the Locust, respectively. If we assume that 10% of this incident field would be evenly distributed over the frequencies above 6 GHz, the *P*_*abs*_ increases to 2.6 nW, 7.7 nW, 14 nW, and 1.0 *μ*W, for the Australian Stingless Bee, the Beetle, the Honey Bee, and the Locust, respectively. These are increases of 370%, 290%, 240%, and 3%, respectively. Note that this is a conservative estimation of the increase in *P*_*abs*_, since we assume a constant incident field and a uniform distribution of the currently used frequencies <6 GHz. Nowadays, most of the incident power density used for telecommunication is located at frequencies ≤2 GHz^1^, where all insects show a minimal *P*_*abs*_. In an isolated approximation (no convection or conduction) and under the assumption of unchanging mass and specific heat capacitance, the rate of temperature increase scales linearly with increasing *P*_*abs*_. As an example, for the Australian Stingless Bee (mass = 2.5 mg) a *P*_*abs*_ of 3 × 10–8 W is estimated for an incident field strength of 1 V/m at 60 GHz. Under the assumption that the insect has a specific heat capacity equal to that of water (4179 J/K kg^43^), this RF-EMF exposure would result in a temperature increase of 3 × 10–6 K/s, in an isolated approximation.

## Strengths and Limitations

Our paper has several clear strengths and contributions to the state of the art in literature. To our knowledge, this is the only paper in which real insects are used to create models for numerical simulations. Moreover, this is the first paper that investigates the exposure of electric fields with RF frequencies associated with 5 G wireless communication and that shows that the absorbed power in insects is expected to increase in unchanged environmental conditions with respect to the one of current wireless communication systems (3 G and 4 G). A disadvantage of our study is the use of homogeneous models in the simulations, whereas real insects will have heterogeneous tissue parameters. Variations on dielectric parameters can exist on a scale that is smaller than the spatial resolution that any scanning method can currently obtain^44^. The FDTD method requires a division of the simulation domain in a number of voxels, which each have to be assigned homogeneous dielectric properties^39^. Any numerical simulation will be an approximation of reality. To our knowledge, the FDTD method, although faced with uncertainties^3,39,44^ is the best simulation method currently available to estimate the quantities studied in this manuscript. This paper is limited to electromagnetic dosimetry, which is focused on determining absorbed powers values. These can be used as an input in thermal modelling of the insects. However, a full thermal analysis was outside the scope of this paper. Finally, we have included variations in angles and polarizations of incident waves. However, we have only looked at a limited number of plane waves, whereas real exposure is composed of plane waves from any direction.

## Future Research

In our future research, we would like to model more insects to get a better understanding of the frequency dependence of the absorbed RF-EMF power as a function of insect size. We would also like to develop heterogeneous insect models with tissue-specific dielectric parameters. Finally, our goal is to determine the effect of absorption of RF-EMFs on the core temperature of insects as a function of frequency. To this aim, we want to use infrared temperature measurements of insects exposed to high electromagnetic fields as function of frequency.

## Conclusions

We investigated the absorbed radio-frequency electromagnetic power in four different real insects as a function of frequency from 2–120 GHz. Micro-CT imaging was used to obtain realistic models of real insects. These models were assigned dielectric parameters obtained from literature and used in finite-difference time-domain simulations. All insects show a dependence of the absorbed power on the frequency with a peak frequency that depends on their size and dielectric properties. The insects show a maximum in absorbed radio frequency power at wavelengths that are comparable to their body size. They show a general increase in absorbed radio-frequency power above 6 GHz (until the frequencies where the wavelengths are comparable to their body size), which indicates that if the used power densities do not decrease, but shift (partly) to higher frequencies, the absorption in the studied insects will increase as well. A shift of 10% of the incident power density to frequencies above 6 GHz would lead to an increase in absorbed power between 3–370%. This could lead to changes in insect behaviour, physiology, and morphology over time due to an increase in body temperatures, from dielectric heating. The studied insects that are smaller than 1 cm show a peak in absorption at frequencies (above 6 GHz), which are currently not often used for telecommunication, but are planned to be used in the next generation of wireless telecommunication systems. At frequencies above the peak frequency (smaller wavelengths) the absorbed power decreases slightly.
